# Do provider birth attitudes influence cesarean delivery rate: a cross-sectional study

**DOI:** 10.1186/s12884-018-1756-7

**Published:** 2018-05-29

**Authors:** Emily White VanGompel, Elliott K. Main, Daniel Tancredi, Joy Melnikow

**Affiliations:** 1 Department of Family Medicine, The University of Chicago, Pritzker School of Medicine, NorthShore University HealthSystem Research Institute, 1001 University Place, Evanston, IL 60201 USA; 2 Department of Obstetrics and Gynecology, The University of Chicago, Pritzker School of Medicine, NorthShore University HealthSystem Research Institute, 1001 University Place, Evanston, IL 60201 USA; 3 California Maternal Quality Care Collaborative, Stanford University, Stanford Medical School Office Building, 1265 Welch Road, MS 5415, Stanford, CA 94305 USA; 4 Center for Healthcare Policy and Research and Department of Pediatrics, University of California Davis School of Medicine, 2103 Stockton Blvd, Sacramento, CA 95817 USA; 5 Center for Healthcare Policy and Research and Department of Family and Community Medicine, University of California Davis School of Medicine, 2103 Stockton Blvd, Sacramento, CA 95817 USA

**Keywords:** Cesarean section, Provider attitudes, Quality improvement, Primary cesarean, Culture

## Abstract

**Background:**

When used judiciously, cesarean sections can save lives; but in the United States, prior research indicates that cesarean birth rates have risen beyond the threshold to help women and infants and become a contributor to increased maternal mortality and rising healthcare costs. Healthy People 2020 has set the goal for nulliparous, term, singleton, vertex (NTSV) cesarean birth rate at no more than 23.9% of births. Currently, cesarean rates vary from 6% to 69% in US hospitals, unexplained by clinical or demographic factors. This wide variation in cesarean use is also seen among individual providers of intrapartum care. Previous research of birth attitudes found providers of intrapartum care hold widely differing views, which may be a key underlying factor influencing practice variation; however, further study is needed to determine if differences in attitudes are associated with differences in clinical outcomes. The purpose of this study was to estimate the association between individual provider attitudes towards birth and their low-risk primary cesarean rate.

**Methods:**

Four hundred providers were drawn from a stratified random sample of all California providers of intrapartum care in 2013 and surveyed for their attitudes towards various aspects of labor and birth. Providers’ NTSV cesarean birth rates were obtained for 2013 and 2014. Covariates included gender, years of experience, practice location, and primary hospital’s NTSV cesarean rate. We used adjusted multivariate Poisson regression to compare cesarean rates and linear regression to compare attitude scores of providers meeting versus not meeting the Healthy People 2020 (HP2020) goal.

**Results:**

Two hundred nine total participants (obstetricians, family physicians, and midwives) completed surveys, of which 109 perform cesareans. Providers’ NTSV cesarean rate was significantly associated with their composite attitudes score [IRR for each one-point increase 1.21 (95% CI 1.002–1.45)]. Physicians meeting the HP2020 goal held attitudes which were significantly more favorable towards vaginal birth: mean 2.70 (95% CI 2.58–2.83) versus 2.91 (95% CI 2.82–3.00), *p* < 0.01.

**Conclusions:**

Provider attitudinal differences are associated with NTSV cesarean rates. Those meeting the HP2020 goal hold attitudes more favorable towards vaginal birth. These findings may present a modifiable target for quality improvement initiatives to decrease low risk primary cesareans.

## Background

Cesarean sections, when used judiciously, save lives; however, research indicates that the use of cesareans in the United States has risen well above the level of necessity and has become a contributor to maternal morbidity and mortality [1–5]. Women who are nulliparous, at full term, with a singleton pregnancy in vertex presentation (NTSV) have been established as a standard population and used as a target group for reducing the cesarean birth rate [6–8]. Healthy People 2020 has set the goal for NTSV cesarean rate at no more than 23.9% of births [6]. Currently, cesarean rates vary from 6% to 69% in US hospitals [9]. Substantial variation persists after adjustment for hospital demographics, referral categories, or teaching status, and after adjustment for patient clinical or sociodemographic factors [9, 10].

Wide variation is also seen between providers, even those practicing within the same hospital and utilizing a laborist model [11]. Over 90% of the variation in the NTSV cesarean rate is due to two indications: “fetal intolerance of labor” and “failure to progress”; indications requiring subjective decision-making by the intrapartum provider [12]. The American Congress of Obstetricians and Gynecologists’ Committee Opinion on limiting intervention during labor highlights the labor management techniques that, despite prevailing evidence, vary significantly among providers [13]. Evidence is filtered through the lens of a provider’s experiences and attitudes [14]; yet how these attitudes affect clinical outcomes has not been evaluated.

Previous research found differences in birth attitudes between providers of different disciplines (obstetrics, family medicine, midwifery) [15, 16]; yet did not examine clinical outcomes. The objective of this study was to examine the association of individual provider NTSV cesarean rates with their attitudes towards birth. In contrast to earlier studies examining physician demographic factors [17–19], this study sought to evaluate a potentially *modifiable* personal attribute that may contribute to cesarean overuse.

### Aim

Our primary aim was to assess the association between providers’ birth attitudes and their NTSV cesarean rates.

## Methods

### Design

A stratified random sample of providers listed as delivering clinicians on California birth certificates in 2013 were surveyed with a previously validated survey instrument of provider birth attitudes [16]. We linked providers’ scores on this survey with their individual NTSV cesarean rates for the years of 2013 and 2014, as calculated by the California Maternal Data Center (CMDC), a service of the California Maternal Quality Care Collaborative. Baseline demographics and survey scores were analyzed using SAS Version 9.4. Poisson regressions were performed using Stata 12.1. This study was approved under expedited review by University of California Davis Internal Review Board. Participants received the consent document, but signed consent was waived by the IRB.

### Setting

California has approximately 3500 providers that are listed as delivering clinicians in a given year; these include obstetricians (OB), Maternal-Fetal Medicine specialists, family medicine physicians, Certified Nurse Midwives (CNM), and California Licensed Midwives (LM). Practice settings are diverse, and include rural, urban, and frontier geographic locations, teaching hospitals, community hospitals, and tertiary care centers. Provider mix at each hospital may include only obstetricians or family physicians, or may include Maternal-Fetal Medicine specialists or midwives. The CMDC combines existing datasets, including monthly discharge and clinical data, birth certificate data, and semi-annual patient discharge data from the California Office of Statewide Health Planning and Development, to create hospital and physician-level quality improvement metrics. We obtained a stratified random sample from 2013 - the latest available year of complete data. At the time of final data collection, 2014 complete data became available, thus we included provider and hospital metrics from both 2013 and 2014 combined. Our primary sample size consideration was based on the six domain scores’ ability to account for variation in NTSV cesarean delivery rate. Using pilot data, we determined that a sample size of 116 patients would provide 80% power to detect an 11.1% partial R-square. To account for intermittent missing variables, we targeted a sample of 130 survey participants.

Inclusion criteria for providers were: having been listed on a birth certificate as the delivering clinician in the year 2013, belonging to one of the key study disciplines (maternal-fetal medicine, OB, family medicine, CNM, LM), and having performed at least 10 deliveries per year. Providers with a license address outside the state of California and those without an identifiable discipline based either on license number prefix or NPI taxonomy code were excluded from sampling.

Based on prior research pointing to important influences on provider practice [17–19], stratification was performed based on three variables: provider discipline (maternal-fetal medicine, OB, family medicine, CNM, LM), geographic location as defined by the federally designated Medical Service Study Areas (rural = population density < 250 persons/square mile; frontier = population density < 11 persons/square mile; urban = anything not rural or frontier) [20], and years in practice (< 5 years, 5–15 years, 16–25 years, > 25 years). The stratified random sample was drawn in two rounds of 200, for a total of 400 sampled providers. To ensure adequate power for analysis, oversampling was performed on provider groups other than obstetricians (who perform the vast majority of all births in California). Researchers were blinded to individual providers’ cesarean rates during sample selection.

### Attitudes survey

The birth attitudes survey, previously validated by Klein and colleagues, included 9 different domains assessing provider attitudes towards different aspects of labor and birth [16]. Six of these domains, comprising 31 Likert-style items, were chosen as those most likely to have an effect on the targeted outcome – low-risk primary cesareans. They included (renumbered for this study): Domain 1: attitudes regarding use of electronic fetal monitoring (Cronbach alpha [α] = 0.704), Domain 2: factors that increase cesarean rates (α = 0.810), Domain 3: fears of birth mode by respondents or their partners/spouses (α = 0.929), Domain 4: factors that decrease cesarean rates (α = 0.819), Domain 5: maternal choice and mothers’ roles in birth (α = 0.646), and Domain 6: safety by mode or place of birth (α = 0.748). The composite scale combined the individual domain scores. Coding of domains 4 and 5 was reversed for directional consistency to create a total mean score. Lower scores on the composite scale indicate attitudes more favorable toward vaginal birth, while higher scores indicate attitudes that favor cesarean birth. Providers were also asked to give their discipline and years of experience. Each provider was assigned a random identifier, so that survey responses were not associated with provider name.

We used best practices for achieving maximal survey response as detailed by Dillman et al. [21] Survey data collection began in October 2015 and ended in April 2016. We sent the attitudes survey via postal mail and included a web address for optional completion online. The initial mailing included the 4-page survey, a cover letter, informed consent document, a self-addressed stamped envelope, and an incentive of a $10 Starbucks gift card. Providers could opt to provide their email addresses to be entered into a drawing at the conclusion of the study for an iPad of approximately $400 value at the conclusion of the study. One to two weeks after the initial mailing, we sent a postcard reminder to complete the survey. Between one and 2 months after the initial mailing, all non-responders were sent a second complete packet including cover letter, consent, survey document, and self-addressed stamped envelope.

### Provider-level cesarean rate

The CMDC database uses the standard NQF-endorsed algorithm [4] to calculate the total number of births attended by a provider that qualify as primary low-risk cesareans. This criteria includes all nulliparous, term (> 37 weeks gestation), singleton gestations in vertex presentation at delivery. These are termed NTSV births for nulliparous, term, singleton, and vertex, and remain the primary target for quality improvement initiatives to decrease cesarean section overuse in the United States [6–8]. We obtained both the total number of NTSV births providers attended in 2013–2014, and the total number of those births that were delivered via cesarean from the CMDC database.

Not all providers included in the survey sample had privileges to perform cesareans. In order to provide a complete picture of the spectrum of attitudes held by all providers practicing as independent clinicians in California, we chose to include all those listed as delivering clinicians on at least 10 deliveries per study year. As it would not be appropriate to assess provider-level cesarean rates in providers who cannot perform cesareans, we excluded these providers from the NTSV predictive model. Both CNMs and LMs do not perform cesareans under any circumstances. A majority of family medicine physicians who do prenatal and intrapartum care do not perform cesareans; however, there are family medicine physicians who have done additional training to qualify to perform cesareans. There is no central database that tracks these privileges, thus this was ascertained by anonymously calling individual family medicine physicians’ offices.

### Covariates

Provider demographic data, including discipline (maternal-fetal medicine, Average Risk OB, family medicine, CNM, LM), gender, years since graduation from medical school (< 5 years, 5–15 years, 16–25 years, > 25 years), practice geographic location (rural/urban), and hospital-level demographics including hospital-level NTSV rate, neonatal intensive care unit level, and percent Medicaid were obtained from the CMDC database. Some providers practiced at multiple hospitals; however, the hospital where they had their greatest number of NTSV births was assigned as their primary hospital of delivery.

### Statistical methods

Due to the large range of providers’ total NTSV birth volume, we used multivariate Poisson regression to maximize precision of our estimation model, using counts of NTSV cesarean deliveries per provider as the dependent variable and total deliveries per provider as an “exposure” variable whose log-transformed value is included as an offset term, to account for between-provider variation. We used robust standard error estimators to protect against model misspecification. Simple regression with predicted margins was used to compare attitudinal scores between discipline groups. Attitude domain and composite means were calculated from all non-missing items. For the dichotomous Healthy People 2020 provider comparison, we compared providers’ attitudes based on whether or not their NTSV cesarean rates met the Healthy People 2020 (HP2020) goal of less than 23.9% [6]. We used multiple linear regression, adjusting for provider gender, practice geography, and experience level to compare predicted means for each group. In order to adjust for the provider’s hospital cultural environment, we adjusted each regression model for the primary hospital’s NTSV rate *exclusive* of provider’s contribution to this rate.

## Results

We received 209 completed surveys, a total response rate of 52.3%, including 22 maternal-fetal medicine, 101 OB, 53 family medicine, 16 CNM, and 17 LM responding. There was a higher response rate for midwives, 97% of who were female, but no difference in disciplinary distribution or gender of physician respondents versus non-respondents. Experience level, practice geographic location, and primary hospital nursery level did not vary between respondents versus non-respondents. Respondents’ primary hospitals had higher annual birth volumes than non-respondents’ hospitals (mean = 5,203.9 versus 2,666.8, *p* < 0.001). Hospital NTSV rates were slightly lower for respondents compared with non-respondents (25.3% versus 26.8%, *p* < 0.01). (Table 1).

**Table 1:** Demographic and Practice Characteristics of Responders and Non-responders

	Responders (n)	Percent	Non-Responders (n)	Percent	*p*-value
N	209		191		
Discipline					*0.35* ^*a*^
MFM	22	10.5	19	9.9	
OB	101	48.3	120	62.8	
Family Medicine	53	25.4	46	24.1	
CNM	16	7.7	3	2.1	
LM	17	8.1	3	1.6	
Gender					*0.29* ^*a*^
Female	132	63.2	102	53.4	
Male	77	36.8	89	46.6	
Experience					*0.49*
< 5 years	35	18.3	28	13.9	
5–15 years	58	30.4	70	34.7	
16–25 years	46	24.1	43	21.3	
> 25 years	52	27.2	61	30.2	
Hospital NICU Level					*0.64*
Basic Nursery	50	23.9	57	29.8	
Community Nursery	82	39.2	73	38.2	
Intermediate Nursery	36	17.2	33	17.3	
Regional Nursery	20	9.6	25	13.1	
Continuous Variables	Mean	SD	Mean	SD	p-value
Hospital Birth Volume^b^	5203.9	3835.9	2666.8	2012.8	< 0.0001
Hospital NTSV CS Rate^b^	25.30	4.80	26.80	6.10	< 0.01

*Abbreviations*: *MFM* maternal fetal medicine, *OB* obstetricians, *Family Medicine* family medicine physicians, *CNM* certified nurse midwives, *LM* licensed midwives, *NICU* neonatal intensive care unit, *NTSV CS* nulliparous, term, singleton, vertex cesarean section

^a^*P*-value includes physicians only. Midwives were all female except for one participant and had exceptionally high response rate

^b^Calculated for provider’s primary delivering hospital site

**Fig. 1:**
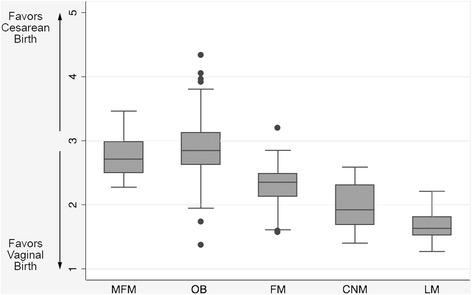
Mean Provider Attitudes Scores on the Composite Scale by Provider Discipline. Figure 1 is a boxplot of providers’ mean attitude scores on the composite attitudes scale categorized by provider membership in a training discipline. Abbreviations: MFM: maternal fetal medicine; OB: obstetricians; family medicine: family medicine physicians; CNM: certified nurse midwives; LM: licensed midwives

**Fig. 2:**
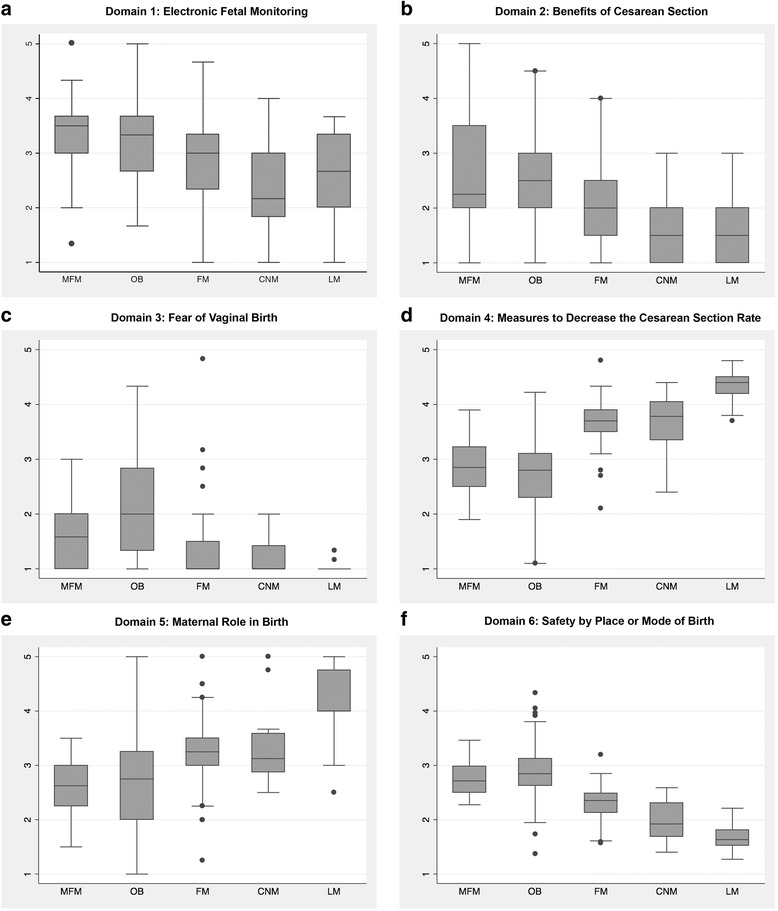
**a**-**f** Boxplots of Provider Attitudes Scores on the Individual Domain Scales by Provider Discipline. Figure 2 **a**-**f** includes individual boxplots of providers’ mean attitude scores for each individual attitudinal domain categorized by provider membership in a training discipline. Abbreviations: MFM: maternal fetal medicine; OB: obstetricians; family medicine: family medicine physicians; CNM: certified nurse midwives; LM: licensed midwives

Attitudes varied significantly according to provider discipline (Fig. 1). Each domain displayed a spectrum of provider attitudes towards birth, with midwives on one end of the spectrum and OBs on the other (Fig. 2). Family medicine physicians either fell between the two or held attitudes more similar to midwives. Maternal-fetal medicine physicians held attitudes most consistent with average risk OBs, but tended to express attitudes that fell between average risk OBs and family medicine physicians. The OB group had widest variation in composite attitude scores (range = 1.37 to 4.33).

Of the 53 responding family medicine physicians, only 8 had privileges to perform cesareans, though 22 had “first assist” privileges. Family medicine physicians with cesarean privileges (family medicine-CS) were identical to family medicine physicians without privileges on the composite scale (mean 2.31 with SD 0.16, and 2.31 with SD 0.35, respectively, *p* = 0.98). For the most part, family medicine-CS physicians expressed attitudes on the individual domains that were slightly less favorable towards vaginal delivery than family medicine physicians; however, family medicine-CS tended to endorse *less* fear of vaginal delivery than their family medicine counterparts, though this was not statistically significant (mean 1.19 ± 0.30 versus 1.40 ± 0.74, respectively, *p* = 0.43). The database attributed cesarean deliveries to 14 family medicine physicians without any cesarean privileges and 10 family medicine physicians with first assist privileges.

The analyses of association of provider attitudes with NTSV cesarean rates included only providers with cesarean privileges and at least 20 NTSV deliveries within the 2 year study period: 11 maternal-fetal medicine, 91 OB, and 7 family medicine physicians. The composite attitudes scale scores for these providers ranged from 1.73 to 4.33 (mean 2.83, standard deviation 0.48). Adjusted incidence rate ratios for domain and composite scale from the Poisson multiple regression are shown in Table 2; this model adjusted for provider gender, practice geography, experience level, and primary hospital’s NTSV rate (exclusive of provider’s contribution to this rate). The composite attitudes scale had an incident rate ratio of 1.21 (95% CI 1.002–1.45, *p* = 0.048), indicating that, for every 1 point increase in a provider’s score on the composite scale, their NTSV cesarean rate *decreased* relatively by 21%. For individual domains, attitudes towards the perceived benefits of cesarean and fear of vaginal birth approached significance [respectively, 1.07 (95% CI 0.99–1.17) and 1.08 (95% CI 0.99–1.17)]. (Table 2).

**Table 2:** Adjusted^a^ Associations between Attitude Scores and Provider NTSV CS Rate^b^

Domain	Incidence Rate Ratio^c^	95% CI	*p*-value
Fetal Monitoring	1.01	0.94–1.10	0.71
Benefits of CS	1.07	0.99–1.17	0.10
Fear of Vaginal Birth	1.08	0.99–1.17	0.09
Measures to Decrease CS^d^	0.91	0.77–1.08	0.28
Maternal Role in Birth^d^	0.93	0.85–1.02	0.14
Safety by Place or Mode of Birth	1.12	0.97–1.30	0.14
Composite Scale	1.21	1.002–1.45	0.048

*Abbreviations*: *NTSV CS* nulliparous, term, singleton, vertex cesarean section, *CS* cesarean section, *CI* confidence interval

^a^Results are from individual Poisson regression models of the NTSV cesarean outcome, with one model for each row, with that variable the focal predictor and with additional covariates used to adjust for gender, experience level, geographic location of practice, primary hospital’s NTSV cesarean rate calculated without the individual provider’s contribution

^b^Only includes providers with confirmed privileges to perform cesarean sections who had at least 20 NTSV births over the two-year study period of 2013–2014

^c^Higher scores indicate attitudes more favorable toward cesarean section except for the two reverse-coded scales (as below)

^d^Higher scores indicate attitudes more favorable toward vaginal birth on these scales

When dichotomizing providers by the Healthy People 2020 NTSV cesarean goal cut-off of 23.9%, our sample reflected the overall California distribution, with 35% of providers meeting this goal and 65% of providers whose rate exceeded the goal. Providers meeting the HP2020 goal held attitudes more favorable toward vaginal birth compared with those over the HP2020 goal [adjusted mean 2.70 (95% CI 2.58–2.83) versus 2.91 (95% CI 2.82–3.00), *p* < 0.01].

## Discussion

We found a significant association between providers’ attitudes and beliefs about birth to their own NTSV cesarean rate. This study was consistent with the findings of the original Canadian survey validation study [16], finding that California providers’ attitudes towards birth were primarily divided along disciplinary lines. One of the most surprising findings in the disciplinary analysis was the wide range of obstetricians’ attitudes, spanning views more pro-cesarean than maternal-fetal medicine providers all the way to those consistent with midwives. This large variation within a single discipline suggests that there may be key acculturation differences in obstetric training and practice environment, where a provider’s attitudes and beliefs are influenced.

We took prior work an important step further by connecting provider attitudes to their own measured clinical outcomes. For providers with cesarean privileges, those meeting the Healthy People 2020 goal held attitudes that were more favorable toward vaginal birth than those not meeting this goal. In our regression analysis, as attitudes became more favorable toward cesarean and less favorable toward vaginal birth, a provider’s NTSV cesarean rate increased proportionately. Additionally, we used a novel adjustor to account for local hospital culture – the primary hospital’s NTSV cesarean rate less the provider’s contribution to that rate. This adjustment highlights the impact of individual provider attitudes on top of that of local hospital norms and practices. These findings suggest that a provider’s underlying attitudes, values, and beliefs play an important role in intrapartum decisions that ultimately affect birth outcomes.

Our results are consistent with, and provide a possible underlying mechanism for, other studies that have found provider differences such as demographics, litigation history, and practice environment are associated with provider self-report personal thresholds to perform cesareans [22, 23]. By independently measuring attitudes while protecting the confidentiality of providers and using administrative data to associate attitudes with an unbiased measure of the individual provider-level cesarean rate, our study minimized social desirability bias, which can confound self-report data.

Of note, the actual magnitude of the point estimate of the association between composite attitudinal score and NTSV cesarean rate is quite large. For every 1 point increase in agreement with attitudes favoring cesarean, NTSV cesarean rates increased by a relative 21%. For a provider with a baseline rate of 25%, this would translate into an absolute change of 5.25%. In comparison, the QUARISMA trial found changes to the absolute cesarean rate of 0.7–2.3% resulting from an audit and feedback mechanism in conjunction with hospital-based best practices implementation [24]. Targeting attitudinal change, which is a “bottom-up” method, in conjunction with quality improvement, or “top-down”, efforts may enhance the impact of interventions. Research is needed to identify effective interventions to enhance evidence-based attitudes towards vaginal birth.

Training may offer a promising target for influencing attitudes that favor vaginal birth. One study found that providers who trained in hospitals with lower obstetric complication rates continued to have lower complication rates once in practice [25]. Most recently, one hospital program significantly decreased their primary cesarean rate by providing senior obstetric supervision of residents on labor and delivery, highlighting the impact of preceptor experience level on trainees [26]. The impact of integrating midwives into traditional obstetric training has been posited but not yet rigorously tested against clinical outcomes [27]. Finally, ongoing training and support after experiencing a traumatic delivery event may mitigate some of the fear attitudes associated with increased cesarean rate, which appear to impact entire hospital units and not just the providers involved. This was described recently in a study of unplanned hospital cesarean rates, which increased and stayed elevated for 4 weeks after any catastrophic neonatal outcome within that hospital [28].

### Limitations

This cross-sectional study cannot draw conclusions regarding causality or time course between independent and dependent variables. Additionally, our a priori sample size justification assumed a linear regression analysis, but we found that a Poisson regression analysis was better suited to the outcome distribution. Thus, in order to assess the adequacy of the realized sample size for the reported effect sizes, one should consider the range of values included within the 95% CI [29]. Where the null value was included along with values that would be clinically meaningful, one could conclude that the effect is ambiguous and would require a more precise estimate in future work. For example, we would assert that a 15% relative increase in the NTSV cesarean rate would be particularly clinically meaningful. When examining the 95% CI for the effect size of the individual attitude items, we find that all of them included the null value (i.e. were not statistically significant) but three of them extended beyond an IRR of 1.15, suggesting that the results for those items are ambiguous and could warrant a study with a larger sample size. For the composite attitude measure, we found statistically significant and clinically significant effects.

Finally, the administrative data used to assess provider-level cesarean rate was restricted to the provider listed on the birth certificate and subject to errors of attribution. For example, the provider listed as “Delivering Clinician” on the Birth Certificate is usually but not always the person that managed the majority of a patient’s labor or made the decision to go to cesarean. Unfortunately, this method discounts information about providers who do not carry cesarean privileges but may play a major role in intrapartum care, and may be key decision-makers along the route that ends in either cesarean or vaginal delivery. For example, a provider may decide to admit a patient prior to the onset of active labor or use continuous electronic fetal monitoring despite a patient’s low-risk status, both of which increase the likelihood of that patient requiring a cesarean, yet the cesarean birth would be attributed to the clinician who performed the surgery itself and not the provider managing the labor. Further studies are needed to assess the interplay of personnel on labor and delivery wards, how key decisions are made and birth outcomes attributed.

## Conclusions

This is the first study we are aware of linking provider attitudinal differences to differences in measured birth outcomes. We found that the more providers’ birth attitudes favored cesarean, the higher their NTSV cesarean rate. In contrast to earlier studies that have focused on physician demographics or litigation history, which are rarely modifiable without major policy changes, we sought to evaluate the influence of a modifiable attribute – a provider’s personal attitudes and beliefs. These findings suggest further scrutiny is needed of how future providers are acculturated during training and while in practice, and how practice groups, hospital units, and inter-professional interactions may modify these attitudes to ultimately improve quality of care and health outcomes.

## Abbreviations

CMDC: California Maternal Data Center
CNM: Certified nurse midwives
family Medicine: Family medicine
family medicine-CS: Family medicine physicians with privileges to perform cesarean
HP2020: Healthy People 2020
LM: California licensed midwives
MFM: Maternal-fetal medicine
NICU: Neonatal intensive care unit
NTSV: Nulliparous, term, singleton, vertex
OB: Obstetrician
